# The House of Charity in Soho

**Published:** 1920-02-14

**Authors:** 


### OUT-OF-THE-WAY INSTITUTIONS.

### The House of Charity in Soho.

It was stated the other day that Marat, the ill-fated revolutionary leader, was at one period of his career a medical man in Soho, his practice being among the poorer class. I happened to spend an afternoon exploring Soho, but I failed to find any traces of Marat. I did find, however, so much historical interest in the region of Soho Square that it seemed to me that I must have passed there scores of times in the past twenty years with my eyes shut.

For example, I saw for the first time the interior of the House of Charity. What a lovely old house this is, and what an interesting history it has! This refuge for stranded men and women was founded in 1846 by Dr. Monro, who was the fifth of his family to practise psychological medicine. He opened the House of Charity in the house described in Dicken's "Tale of Two Cities" as the residence of Dr. Manette and his daughter. His conviction was that many mental cases would never have needed his ministrations if in a time of storm and stress they had had some help to tide them over their difficulties. After ten years the present house in Greek Street was acquired, and here temporarily sick or harassed people who cannot be given accommodation in ordinary hospitals are still received and helped in every way. In many cases the guests have been out-patients at ophthalmic and other hospitals; some have been referred to the House of Charity by the Vigilance Society or the police; all have been men and women who deserved and were grateful for such a friend in need.

Looking round this little-known institution, one found it indeed a sanctuary of rest. It is supposed that once the house was occupied by Alderman Beckford, one of London's merchant princes, and that he was responsible for the ceiling in the Council Chamber, which is a masterpiece, and for the rich carvings which are to be seen everywhere. Going up a curved-rail stairway, specially designed for the ladies who wore the crinoline, one admired the airy, pleasant rooms used by the guests. The latter, of course, are asked to pay nothing, but as a rule they are anxious to give all they can afford towards the funds. The warden, the Rev. H. E. Simpson, says that one day an old lady called and said she wanted to give a present to the House because fifty years before, on being knocked down in the street while on a visit to London, she was given hospitality there. She produced £100 in a bag. The Warden was rather reluctant to take the donation—such things, it is true, are all too rare nowadays—when the old lady cried sharply, "I want the bag back, please, but not the money."

What extraordinary stories cluster about Soho Square! A few yards from the House of Charity is St. Patrick's Church. On this site stood the notorious Carlisle House, occupied by the adventuress, Madame Cornelys, who, after drawing the fashionable folk of the town to her costly entertainments for many years, died in Fleet prison. Across the Square I found the house in which Sir Joseph Bankes, President of the Royal Society, lived with his eccentric and curiously costumed sister, to which came the foremost men of science of his day. It is now stocked from floor to roof with antiques, which with its associations make it doubly interesting. And round the corner I noticed the house in which De Quincey lodged just before he wrote his "Confessions of an Opium Eater." The story of De Quincey's life there, is a sad one, and the dilapidated dwelling, long untenated and now for sale, had a melancholy, forgotten air, in keeping with its history. It is more cheerful to think of the beautiful music at St. Anne's, Soho, or the manufacture of appetising condiments, or the tremendous growth of the cinema industry. The district is not the Bohemian centre it used to be. Amid so many changes I rejoice in the abiding usefulness of the old House of Charity, and I trust its work of healing will continue for many years to come.

